# Exploring the sound environment in neonatal intensive care units as perceived by medical professionals

**DOI:** 10.20407/fmj.2025-008

**Published:** 2025-08-06

**Authors:** Masato Sugiura, Jun Shimizu, Kazuteru Niinomi

**Affiliations:** 1 Faculty of Nursing, School of Health Sciences, Fujita Health University, Toyoake, Aichi, Japan; 2 Faculty of Global Nursing, Otemae University, Osaka, Osaka, Japan; 3 Department of Integrated Health Sciences, Graduate School of Medicine, Nagoya University, Nagoya, Aichi, Japan

**Keywords:** Neonatal intensive care unit, Preterm infants, Medical professionals, Sound environment, Noise

## Abstract

**Objectives:**

This study aimed to identify specific care scenarios and procedures perceived as noisy or quiet by medical professionals working in the neonatal intensive care unit (NICU) and elucidate factors influencing the sound environment that may affect preterm infants.

**Methods:**

Semi-structured interviews were conducted with 12 nurses and eight doctors working in the NICU. Participants identified care and procedural situations perceived as noisy or quiet for preterm infants. Specific sound environment factors that could potentially affect preterm infants were extracted and visualized to demonstrate the interrelationships among these factors.

**Results:**

Medical professionals recognized that noise levels fluctuated based on specific care scenarios, with sudden sounds and background noises amplifying disturbances in otherwise quiet settings. Participants also identified the overlap of multiple noise sources in various locations, including continuous beeping or other disruptive noises, as potentially affecting preterm infants.

**Conclusion:**

Because noise levels in the NICU fluctuate during specific care scenarios, comparative verification of noise levels inside and outside the incubator is necessary. The present results revealed that even in periods perceived as quiet, sudden sounds and background noises may amplify disturbances to preterm infants. Furthermore, because overlapping noise sources during care and procedures affect the sound environment, empirical evaluation of sound quality by measuring noise during quiet hours is recommended.

## Introduction

Noise in the neonatal intensive care unit (NICU) is a source of stress for preterm infants and remains a prevalent issue in this environment.^1,2^ Incubators expose preterm infants to noise sources such as motor sounds, opening and closing of doors, and alarms from medical equipment. Moreover, the NICU environment, including the area around the incubator, influences noise levels inside the incubator. Incubators provide limited acoustic isolation, and external noise can easily penetrate and affect the infant’s auditory environment. These combined factors make it challenging to create a sound environment that is comfortable and soothing for preterm infants.

Most studies that evaluated such environments focused on the loudness of the sound sources. The principal evaluation indices in those studies were physical properties, such as noise levels to guide reduction efforts. The American Academy of Pediatrics stipulated 45 dB as the upper noise limit that a preterm infant can tolerate, which prompted the implementation of measures to keep the noise level below this threshold.^3^ Specifically, traditional noise reduction approaches in the NICU have involved conducting noise level assessments from sound sources in NICU rooms.^4^ These noise level assessments primarily focused on determining whether sound levels were high or low. However, they overlooked the dynamic sound environment associated with preterm infant care, especially given the round-the-clock procedures performed in the NICU. Beyond noise intensity, numerous factors related to the sound environment and medical care may influence preterm infants’ responses. These factors include the duration and variability of noise in the NICU and inside the incubator, as well as the care and procedures administered by medical professionals.^5–7^

Measuring NICU noise levels is challenging as various caregiving activities mean the levels constantly fluctuate. This makes standardized measurements difficult to interpret. Objective measurements alone cannot capture how medical professionals perceive the nuanced effects of noise on preterm infants, who are influenced by contextual factors such as the timing and intensity of sounds relative to caregiving activities. Preterm infants’ responses to noise are often subtle and require observation over time. Therefore, real-time assessment of noise impact is virtually impossible through technical measurements alone. Understanding the perceptions of medical professionals, who observe infant responses and identify noise-generating scenarios daily, is essential before attempting comprehensive noise level measurements.

Despite previous efforts, a significant challenge remains for NICU medical professionals in that they lack a comprehensive understanding of the specific care activities and environmental factors that most significantly contribute to noise disturbances for preterm infants. This limited knowledge makes it difficult for medical professionals to implement targeted noise reduction strategies during their daily practice. Furthermore, although objective measurements of sound levels are valuable, they cannot fully capture the complex real-world situations in the NICU environment, including infants’ subtle responses to various sound stimuli that experienced medical professionals observe during daily care.

This study focused on the various caregiving and procedural scenarios that are conducted around the clock by medical professionals in the NICU. We aimed to identify specific situations that medical professionals recognized as causing noise or contributing to a quiet environment. Understanding both noisy and quiet situations allowed us to identify factors that generated noise and those that promoted a calm auditory environment. Such knowledge will facilitate environmental adjustments for preterm infants’ physiological stability and development. Saito et al.^8^ recently conducted a questionnaire survey investigating noise perception among medical professionals and reported on sound levels inside the incubator perceived as noise. However, that study primarily addressed noise attributed to sudden sounds and only analyzed specific short periods, which limited its applicability to the NICU environment. Care interventions for preterm infants require tailored approaches, and it is difficult to determine the characteristics of the sound environment using surveys that only measure sudden sounds generated in brief periods. Such methods do not adequately capture noise fluctuation patterns or medical professionals’ perceptions of the sound conditions.

A key challenge is identifying the specific care activities and environmental factors perceived by medical professionals as contributing to problematic noise levels for preterm infants. This study used semi-structured interviews with experienced NICU medical professionals to gather detailed information about actual noise conditions and their variations in clinical settings that could not be captured through quantitative measurements alone. These professionals are in a frontline position to observe subtle patterns in infants’ responses to various sound stimuli and recognize the care activities that consistently generate problematic noise levels. Identifying specific scenarios and situations perceived as noisy or quiet is crucial before measuring noise levels inside the NICU or incubator.

Therefore, this study aimed to address the following research questions. (1) What specific caregiving and procedural scenarios are perceived as noisy or quiet by medical professionals working in the NICU, including their impact on the auditory environment in the incubator? (2) What factors in the NICU sound environment, as identified by medical professionals, potentially affect preterm infants? (3) How do these identified factors interrelate to form the dynamic sound environment experienced by preterm infants? By answering these questions, this study provided insights to guide the creation of a comfortable auditory setting for preterm infants.

## Methods

### Study design

This study employed a qualitative descriptive approach with data collected in semi-structured interviews.

### Study participants

Participants comprised 20 medical professionals: 12 NICU nurses and eight pediatric/perinatal doctors. The mean work experience was 5.2 years for nurses and 12.8 years for doctors (Table 1).

### Data collection

Data were collected from August to October 2022. Participants were selected by the ward manager based on clinical experience. After providing consent, the participants completed semi-structured interviews (20–30 minutes each) on caregiving scenarios perceived as noisy or quiet. Key interview questions addressed noisy and quiet caregiving scenarios and their impact on the NICU environment.

### Data interpretation

Verbatim transcripts were created from the interview audio recordings. Meaningful phrases were extracted and grouped by thematic association with caregiving scenarios and noise perception. Instead of applying a hierarchical coding structure, categories were directly developed to reflect medical professionals’ perceptions (Tables 2–4). The relationships among these categories and related factors were visually represented to illustrate the final derived associations.

### Ethical considerations

This study was approved by the Ethical Review Committee of Fujita Health University (HM22-247). All participants provided written consent.

## Results

### Sources of noise affecting preterm infants

Six main noise sources were identified (Table 2).

### Caregiving scenarios and procedures perceived as noisy

In addition to specific sound sources, medical professionals identified broader clinical situations with increased noise, including admission procedures, postural care, X-ray imaging, and busy visiting hours (Table 3).

### Caregiving scenarios and procedures perceived as quiet

Quiet scenarios included post-feeding, nighttime with dimmed lights, and kangaroo care (skin-to-skin contact between a parent and infant) (Table 4).

### Factors likely to affect the sound environment

**Figure 1:**
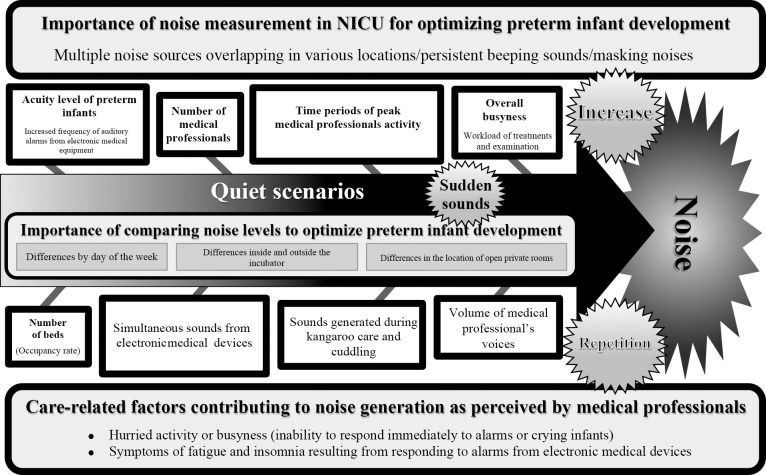
Factors associated with the sound environment affecting preterm infants in the neonatal intensive care unit (NICU) 1. The square boxes linked to the arrows indicate factors identified by medical professionals that can lead to sudden increases in noise during otherwise quiet scenarios in the NICU, potentially affecting preterm infants’ well-being. These factors include infant acuity level, number of medical professionals, peak activity times, occupancy rates, workload and busyness, volume of electronic device alarms, noise during kangaroo care, and loudness of medical professionals’ voices. 2. Further analysis found that these noise-contributing factors differed by day of the week, location of open private rooms, and sound levels inside/outside incubators, which impacted the increase in noise levels as shown by the arrows. 3. The star shapes represent medical professionals’ recognition that sudden sounds, increases in volume, and repetitive noises during quiet periods can raise noise levels, thereby negatively affecting preterm infants. 4. Persistent and overlapping alarms from medical devices in various locations were perceived by medical professionals as masking each other and contributing to excessive noise. In turn, this was linked to hurried activity, delayed responses to alarms or crying infants, and symptoms of fatigue and insomnia among medical professionals. These findings highlight the importance of noise measurement and comparative analysis for optimizing the NICU environment to support preterm infant development. These care-related factors highlight the specific nursing practices, such as kangaroo care and cuddling, that contribute to noise levels in the NICU, as identified by medical professionals. Understanding these factors is crucial for optimizing the sound environment to support preterm infants’ development.

Factors that potentially disturbed preterm infants in otherwise quiet settings, as identified by medical professionals, were infant acuity, staff numbers, activity peaks, occupancy rates, device alarms, and staff voices. These disturbances varied by day of week, room location, and incubator conditions (Table 5, Figure 1).

Figure 1 summarizes how noise sources (Table 2) interact with caregiving scenarios (Tables 3 and 4) and environmental factors (Table 5) to shape the NICU sound environment. Sudden alarms can disrupt quiet scenarios, and overlapping noise sources may compromise interventions requiring calm. Figure 1 visually represents these relationships.

## Discussion

This study addressed a knowledge gap by exploring how medical professionals in the NICU perceived noise as a physical phenomenon and in relation to caregiving scenarios and procedures. Unlike prior studies that focused on decibel levels, our approach emphasized the contextual and operational factors shaping the sound environment.

The finding that sudden or intermittent sounds during otherwise quiet periods were perceived as disruptive highlighted several key factors contributing to increased noise levels, including: differences in room location, daily variations in personnel numbers, and sound level variations inside versus outside incubators. Noisy procedures included admission procedures, ophthalmic examinations, blood sampling, X-ray imaging, and IV changes, all of which involved multiple staff and equipment. Quiet scenarios included post-feeding periods, nighttime with dimmed lights, and kangaroo care. However, even these periods were vulnerable to sudden sounds that could disrupt the auditory environment for infants inside incubators.

This study identified caregiving scenarios medical professionals perceived as noisy or quiet and clarified key factors contributing to noise. Admission procedures were noisy because of overlapping alarms and human activities, whereas kangaroo care was generally quiet but still susceptible to disruptive sounds. These findings highlighted the context-dependent nature of noise perception, influenced by both human activities and environmental factors. This extended previous research by integrating qualitative insights with established knowledge about noise sources. Previous research identified factors that were similar to our findings, such as bed numbers, staff presence, alarms, and infant acuity.^9^

A previous study suggested that more than 40% of the noise in the NICU is attributed to nursing activities, followed by 30% from human factors (including conversations, laughter, footsteps, and other sounds generated by people), and 20% from equipment alarms.^10^ In this context, “human activities” refer to specific actions by medical professionals (e.g., procedures and movements) that directly generate noise, whereas “human factors” include broader elements such as conversations and other sounds from people in the NICU. When multiple alarms occur simultaneously and prompt responses are difficult, busy and rushed activity may further increase noise levels.

Although noise reduction measures in the NICU are designed to target expected noisy caregiving scenarios and procedures, they may inadvertently generate or increase noise. In this study, medical professionals observed preterm infants’ reactions (e.g., facial expressions and body movements) in response to sustained or sudden sounds. Such noise has been reported to disrupt infants’ sleep and wakefulness levels, leading to behaviors such as agitation, crying, and irritability.^11^ In addition, noise is known to interfere with preterm infants’ physiological stability and sleep patterns^2^ and negatively impact their hearing, growth, and development.^12,13^ Excessive noise, particularly from sudden sounds during sleep, may impose stress on preterm infants. Therefore, creating a quiet environment during rest periods is essential, and requires comprehensive evaluation of sound levels both inside and outside incubators.

The present findings suggest actionable insights for NICU design and operations, such as reducing overlapping alarms during peak times and optimizing room layouts to minimize sound transmission. Understanding staff perceptions enables interventions that balance clinical efficiency with environmental comfort. In the NICU, simply lowering sound intensity may not create a comfortable environment. Medical professionals’ perceptions of noise vary by context; for example, some considered staff conversations noise, whereas others did not view examination discussions as disruptive. Frequent exposure to alarms from medical devices may require nurses to urgently attend to infants, potentially leading to risks such as fatigue and insomnia symptoms.^14^ Because noise affects both infants and staff performance, evaluating sound quality in real clinical settings is essential for creating an optimal acoustic environment.

### Limitations

Sound loudness and pitch may affect clinical work depending on how specific noise levels and frequencies are perceived. This study was conducted at a single institution, and perceptions of the sound environment may vary with facility systems, patient acuity, and staff experience. To enhance the credibility and generalizability of these findings, multi-institutional surveys and direct measurements of noise levels in the NICU and incubators are needed. Further research should comprehensively characterize the NICU sound environment and support the development of strategies to create a comfortable auditory setting for preterm infants.

## Conclusion

This study clarified caregiving scenarios and procedures in the NICU that medical professionals perceived as noisy or quiet, along with associated factors that may affect preterm infants. Understanding these perceptions is essential for evaluating and managing the NICU auditory environment, especially by assessing sound quality both inside and outside incubators during quiet periods.

## Figures and Tables

**Table 1:** Interview participants’ basic information

Nurses			
No.	Gender	Years of nursing experience	Years of NICU experience
1	F	1	1
2	F	2	1
3	F	1	1
4	M	4	3
5	F	4	2
6	F	9	4
7	F	8	5
8	F	10	5
9	F	9	7
10	F	10	9
11	F	17	17
12	F	10	7

Doctors			
No.	Gender	Years of experience as a doctor	Career
13	F	21	Pediatrics and perinatal specialist, supervisor
14	M	17	Pediatrics and perinatal specialist
15	M	15	Pediatrics and perinatal specialist
16	F	13	Pediatrics and perinatal specialist
17	M	10	Pediatrics and perinatal specialist
18	M	9	Pediatrician
19	F	9	Pediatrician
20	M	8	Pediatrician

NICU, neonatal intensive care unit.

**Table 2:** Categories of noise sources that may affect preterm infants as perceived by medical professionals

Category	Representative responses (quotes from interviews)
Alarm sounds, including monitors, ventilators, syringe pumps, and infusion pumps	“Monitor alarms are the most prominent source of noise in the NICU, with ventilator alarms being particularly audible throughout the unit.” (Nurses, Doctors) “Operational sounds from medical devices such as syringe pumps, infusion pumps, and CPAP, as well as alarms indicating completion of infusion or milk feeding, also contribute to noise pollution.” (Nurses, Doctors) “Alarms for abnormal conditions such as decreased oxygen saturation, irregular pulse/respiratory rates, and apnea are essential but constitute a significant source of noise.” (Doctors)
Voice: including conversations and laughter among medical professionals, conversations with family members, and calls for picking up items	“During infant care or interventions, medical professionals may engage in unrelated conversations or discuss other tasks, potentially neglecting priority communication with the baby.” (Nurses) “Conferences, discussions, and shift handovers at the staff station can be loud enough to be heard in adjacent rooms and nearby beds, potentially affecting patients.” (Nurses, Doctors) “During rounds and patient information sharing, multiple medical professionals conversing while moving between bedsides can create audible, though not necessarily disruptive, noise.” (Doctors)
Noise: including sounds of moving trolleys and portable X-ray machines, dropping objects, hitting walls, opening and closing of shelves and trash can lids, removing equipment	“The opening and closing of trash cans at the bedside, as well as the movement of trolleys and carts, are noticeable sources of noise.” (Nurses, Doctors) “Conferences and conversations at the nurse station can be heard loudly even from the bedside.” (Nurses) “Sudden loud noises can occur, such as equipment being dropped or sounds during X-ray procedures.” (Doctors)
Cries: Multiple preterm infants crying and high-pitched crying of preterm infants outside the incubator (cot)	“Just before feeding times, multiple infants may start crying simultaneously, triggering monitor alarms. This situation becomes a significant source of noise, even during night shifts.” (Nurses) “Not only orally fed infants, but also non-intubated infants or those on high-flow oxygen in cots may start crying before milk feeding times.” (Nurses) “The cries of other infants, especially those crying intensely, are a major contributing factor to noise in the unit.” (Nurses, Doctors)
Sound generated by human mobility (footsteps) within the floor and sound of putting on/off gowns	“The rustling sound of removing gowns and footsteps are noticeable even though they are relatively quiet noises.” (Nurses, Doctors) “While these sounds are not particularly loud, they can be noticeable enough to draw attention in the quiet environment of the NICU.” (Nurses, Doctors)
Sound inside incubators: Sounds of opening and closing of care windows and treatment windows, sounds of hands or objects hitting the walls of the incubator, sounds generated when monitor cords or routes are inserted or removed, sounds of inclining the bed table back, sounds of opening and closing of drawers and X-ray cassette trays, and vibration sounds inside the incubator	“Various sounds associated with incubators, such as opening and closing of portholes or large apertures, and objects contacting the walls, can occur and potentially cause stress for the infant.” (Nurses Doctors) “While it’s difficult to directly observe infants inside incubators, those in infant beds or cots are often seen startling or waking in response to these sounds.” (Nurses) “Even relatively small sounds like inserting or removing a stethoscope, opening and closing doors, or changing diapers can be stressful stimuli depending on the infant’s sound perception.” (Doctors)
Opening/closing sound of automatic doors	“In some rooms, the opening and closing sounds of automatic doors can be frequently heard because of their proximity.” (Nurses)

NICU, neonatal intensive care unit; CPAP, continuous positive airway pressure.

**Table 3:** Categories of scenarios or situations involving care and procedures perceived as noisy

Category	Representative responses (quotes from interviews)
Admission procedures: Inpatient transport, blood sampling, intravenous placement, gastric intubation, and tracheal intubation	“During new admissions and emergencies, various sounds occur from procedures, medical equipment, and medical professionals’ conversations, making the entire ward noisy. After admission, procedures such as intravenous placement and intubation involve multiple medical professionals, which tends to generate more noise.” (Nurses, Doctors) “Admission responses and morning care times are especially busy, with increased medical professionals’ movement raising noise levels. However, there tends to be a calmer atmosphere around noon.” (Nurses)
Care provision before the start of procedures, including tube feeding, cleanliness care, changing sheets, changing diapers, environmental maintenance, measuring vital signs, and changing body posture.	“When providing care to infants in incubators, especially those with respiratory support, two medical professionals often work together. The noise level increases due to frequent opening and closing of incubators, crying infants during procedures, and conversations between medical professionals. These interventions are particularly noticeable at night and, combined with monitor alarms, are perceived as noise.” (Nurses)
Procedure and examination: Ophthalmic examinations and blood sampling	“During procedures like blood sampling or ophthalmic examinations, monitor alarms may sound and equipment movement can create noise. When both hands are occupied during procedures, alarm sounds may continue for longer periods.” (Nurses)
X-ray filming	“During X-ray imaging, various sounds are produced when adjusting the infant’s position in the incubator or setting up equipment. Medical professionals try to work quietly, but the noise can be noticeable. When disconnecting heart rate monitors, alarms continue to sound, and this noise may persist during imaging as hands are occupied. Additionally, necessary conversations while the incubator window is open during X-rays can also be a source of noise.” (Nurses)
Visiting hours: Guidance by nurses to family members	“Visiting hours typically coincide with feeding times. During afternoon visiting hours, monitor alarms tend to go off more frequently. Alarms can be triggered when family members hold the baby or when the baby moves, causing the monitor to detach. During day shifts, we perform care with mothers, but during evening shifts, we limit care with mothers because of reduced staffing.” (Nurse)
Handover/notification and rounds: Conference	“During weekday mornings, especially during rounds at 11 AM and morning handovers, there is an increase in medical professionals’ conversations and voices, making the environment noisier. Bedside discussions for confirming instructions and calling out to doctors at a distance may also be potential sources of noise.” (Nurses)
Change of IV drips	“There are many mechanical sounds during IV drip changes and when starting new IVs. Especially for patients requiring multiple three-way stopcocks, we try to be careful not to make noise when operating inside incubators, but it’s difficult to avoid sounds like stopcocks catching. These sounds are relatively less frequent during night shifts.” (Nurse)
Responding to sudden changes, including preparation for procedures	“During emergencies or sudden changes, the noise level increases significantly as medical professionals make calls to family members and additional personnel, creating a sudden increase in activity and noise. While loud sounds can also occur from equipment falling or other incidents, these are relatively infrequent.” (Doctors, Nurses)
Weaning from mechanical ventilation	“Ventilator alarms are crucial for alerting abnormal values, but they can be very disruptive when ringing for long periods or at high volumes. Some ventilator models have particularly loud alarms, and hospital regulations require maintaining a certain minimum volume. This can potentially be uncomfortable for both patients and medical professionals.” (Nurse)

IV, intravenous.

**Table 4:** Categories of scenarios or situations involving care and procedures perceived as quiet

Category	Representative responses (quotes from interviews)
30 minutes to 2 hours after start of tube feeding	“After feeding, babies tend to be satisfied and settle down about 30 minutes to an hour later. During the rest period between feedings, it’s often quiet with few alarms going off.” (Nurses)
Relaxed time after handover (17:00~19:00)	“The period from around 17:00 to 19:00, after the 16:00 feeding ends and before the night shift begins, is relatively quiet compared with other times during the day. This time between the end of the day shift and start of the evening shift is a calmer period. However, it tends to get noisy again around 20:00 during the transition to the night shift.” (Nurses)
When the lights are dimmed at night (22:00~4:00)	“The night shift, especially during the early morning hours, is extremely quiet with dimmed lights. Rooms 4 and 8 in particular become very dark, creating a home-like atmosphere. During this time, there are fewer medical professionals on duty and almost no visitors.” (Nurses, Doctors)
During break time for medical professionals (after 11:00~)	“After care is completed around 10 AM, the period between 11 AM and 1 PM before the next feeding is generally calm. Medical professionals take breaks in shifts, reducing the number of people present, which contributes to the quietness. In contrast, mornings tend to have a busier atmosphere.” (Nurses)
Kangaroo care or cuddling during visiting hours	“During kangaroo care or visiting hours, there’s less medical professionals’ movement and it’s quieter. Babies tend to be calmer. When parents hold them, their heart rates often decrease and monitor alarms are less likely to go off.” (Nurses)
At the time of hearing test	“During hearing tests (ABR), excessive ambient noise can make the examination difficult to perform, causing discomfort and challenges. To ensure a quiet environment, we sometimes need to relocate the test to a different area.” (Doctors)
During feeding times	“Visiting hours are typically scheduled to coincide with feeding times. During these visits, sounds from opening and closing partitions for breastfeeding privacy and conversations with visitors occur. These sounds may be stimulating for the babies.” (Nurses)

**Table 5:** Categories of factors affecting the sound environment during quiet scenarios in the NICU

Categories of NICU sound factors	Sound factor relationships
Acuity level of preterm infants	Higher acuity levels among infants often result in frequent alarms from monitors and more complex procedures requiring prioritization. These factors collectively contribute to a noisier environment, especially during new admissions or emergencies.
Number of medical professionals	An increase in medical professional numbers can lead to overlapping voices and additional noise sources. Conversely, quieter conditions tend to occur during night shifts when fewer personnel are present. These observations suggest a direct relationship between medical professional numbers and noise levels in the NICU environment.
Time periods of peak medical professional activity	Medical professionals engage in simultaneous activities at specific times, particularly around 9 AM, leading to increased voices and noises. These peak activity periods, although necessary for medical care, have the potential to impact the sound environment.
Overall busyness (workload of treatments and examination)	During periods of concentrated activities such as morning care or milk feeding, simultaneous procedures increase the level of noise. Overlapping sounds (e.g., monitor alarms triggered during care) contribute to a noisier environment. This heightened activity is considered a factor that influences the NICU acoustic environment.
Number of beds (occupancy rate)	Higher occupancy rates are associated with increased equipment use and diverse noise sources, potentially raising noise levels. In contrast, lower occupancy rates may allow for quieter conditions. These patterns indicate that bed occupancy influences the acoustic environment.
Simultaneous sounds from electronic medical devices	Overlapping alarms from multiple medical devices can amplify discomfort and mask certain sounds. These overlapping noises create a complex auditory environment that may impact preterm infants’ auditory development.
Sounds generated during kangaroo care and cuddling	Even during typically quiet scenarios such as kangaroo care or nighttime hours, sudden noises can elevate noise levels. Such unexpected sounds are more noticeable because of the contrast with quiet surroundings and may affect preterm infants’ stability.
Volume of medical professionals’ voices	The need for louder communication because of masks or distance can increase voice levels. Furthermore, conversations unrelated to direct infant care may contribute to noise. Such situations can disrupt the calm environment intended for preterm infants.
Differences by day of the week	Weekdays tend to be noisier because of higher activity levels such as rounds and examinations, whereas weekends, particularly Sundays, are quieter with reduced procedures and fewer personnel. This highlights variations in sound environments based on the day of the week.
Differences inside and outside the incubator	Although incubators provide some sound insulation, opening or closing them introduces direct noise exposure. The use of incubator covers enhances sound insulation but remains incomplete. These conditions create varying auditory environments for preterm infants inside and outside incubators.
Differences in the location of open private rooms	Noise propagation varies depending on spatial configurations. Rooms closer to the staff station or surrounded by walls tend to amplify sounds, whereas some areas maintain relatively quiet conditions. These spatial differences influence sound exposure for preterm infants.

NICU, neonatal intensive care unit.

## References

[1] Etzel RA, Balk SJ, Bearer CF, et al. Noise: a hazard for the fetus and newborn. Pediatrics 1997; 100: 724–727. 9836852

[2] Ranganna R, Bustani P. Reducing noise on the neonatal unit. Infant 2011; 7: 25–28.

[3] Almadhoob A, Ohlsson A. Sound reduction management in the neonatal intensive care unit for preterm or very low birth weight infants. Cochrane Database Syst Rev 2015; 1: CD010333. 25633155 10.1002/14651858.CD010333.pub2

[4] Thomas KA, Uran A. How the NICU environment sounds to a preterm infant: update. MCN Am J Matern Child Nurs 2007; 32: 250–253. 17667291 10.1097/01.NMC.0000281966.23034.e9

[5] Smith SW, Ortmann AJ, Clark WW. Noise in the neonatal intensive care unit: a new approach to examining acoustic events. Noise Health 2018; 20: 121–130. 30136672 10.4103/nah.NAH_53_17 PMC6122266

[6] Restin T, Gaspar M, Bassler D, Kurtcuoglu V, Scholkmann F, Haslbeck FB. Newborn Incubators Do Not Protect from High Noise Levels in the Neonatal Intensive Care Unit and Are Relevant Noise Sources by Themselves. Children (Basel) 2021; 8: 704. 34438595 10.3390/children8080704 PMC8394397

[7] Gramajo E. The Effects of Loud NICU Environments on Premature Infants and Interventions to Help Minimize Noise. Nursing|Senior Theses 2023: 1–26.

[8] Saito Y, Kurokawa A, Takahashi N. NICU nai no Otokankyō ni taisuru Ishiki Kōjō e no Torikumi(Efforts to raise awareness of the sound environment in the NICU). The journal of Narita Red Cross Hospital 2014; 16: 64–68 (in Japanese).

[9] Mayhew KJ, Lawrence SL, Squires JE, Harrison D. Elevated sound levels in the neonatal intensive care unit: what is causing the problem? Adv Neonatal Care 2022; 22: E207–E216. 35446264 10.1097/ANC.0000000000000996 PMC10519292

[10] Joo SH, Kim TI. Noise level and frequency experienced by premature infants receiving incubator care in the neonatal intensive care unit. Child Health Nurs Res 2020; 26: 296–308. 35004473 10.4094/chnr.2020.26.2.296 PMC8650934

[11] Thomas KA. How the NICU environment sounds to a preterm infant. MCN Am J Matern Child Nurs 1989; 14: 249–251. 2473369

[12] Wachman EM, Lahav A. The effects of noise on preterm infants in the NICU. Arch Dis Child Fetal Neonatal Ed 2011; 96: F305–F309. 20547580 10.1136/adc.2009.182014

[13] Beken S, Onal E, Gunduz B, Cakir U, Karagoz I, Kemaloglu YK. Negative effects of noise on NICU graduates’ cochlear functions. Fetal Pediatr Pathol 2021; 40: 295–304. 31984823 10.1080/15513815.2019.1710788

[14] Graham KC, Cvach M. Monitor alarm fatigue: standardizing use of physiological monitors and decreasing nuisance alarms. Am J Crit Care 2010; 19: 28–34. 20045845 10.4037/ajcc2010651

